# Diet-Induced Early Inflammatory Response of Visceral Adipose Tissue in Healthy Male Wistar Rats

**DOI:** 10.3390/nu16081184

**Published:** 2024-04-16

**Authors:** Iliyan Dimitrov, Teodora Stankova, Penka Angelova, Nikolay Boyadjiev, Katerina Georgieva, Ivica Dimov, Anelia Bivolarska, Milena Draganova, Fanka Gerginska, Elena Daskalova, Vilian Gramatikov, Slavi Delchev

**Affiliations:** 1 Department of Medical Biochemistry, Faculty of Pharmacy, Medical University of Plovdiv, 4002 Plovdiv, Bulgaria; teodora.stankova@mu-plovdiv.bg (T.S.); ivica.dimov@mu-plovdiv.bg (I.D.); anelia.bivolarska@mu-plovdiv.bg (A.B.); 2 Department of Physiology, Faculty of Medicine, Medical University of Plovdiv, 4002 Plovdiv, Bulgariakaterina.georgieva@mu-plovdiv.bg (K.G.); 3 Department of Medical Biology, Faculty of Medicine, Medical University of Plovdiv, 4002 Plovdiv, Bulgaria; milena.draganova@mu-plovdiv.bg; 4 Research Institute, Medical University of Plovdiv, 4002 Plovdiv, Bulgaria; 5 Department of Human Anatomy, Histology and Embryology, Faculty of Medicine, Medical University of Plovdiv, 4002 Plovdiv, Bulgaria; fanka.gerginska@mu-plovdiv.bg (F.G.); elena.daskalova@mu-plovdiv.bg (E.D.); slavi.delchev@mu-plovdiv.bg (S.D.); 6 Faculty of Medicine, Medical University, 4002 Plovdiv, Bulgaria; vilian2007@gmail.co.uk

**Keywords:** low-grade inflammation, visceral adipose tissue, high-fat diet, animal model, CRP, SAA, IL-4

## Abstract

The prolonged consumption of a high-fat diet (HFD) leads to abnormal growth of the visceral adipose tissue (VAT), increased macrophage infiltration, and altered secretion of biologically active molecules. This is considered as a precondition for the development of obesity, inflammation, and obesity-related disorders. Therefore, we studied HFD-induced changes in the tissue levels of the inflammatory markers C-reactive protein, serum amyloid-A, and interleukin-4 in healthy male Wistar rats. The animals were first divided at random into two groups subjected to either a standard or a high-fat diet. The initial effect of the diet was evaluated after fourteen weeks. In order to study the diet duration effect, the standard diet was given to twelve animals from the HFD group, while the remaining continued with the HFD for an additional four weeks. Our results showed that the HFD barely affected body mass index, conicity, relative fat mass, and Lee indices, whereas it provoked adipocyte hypertrophy and gradually increased the levels of both the pro- and anti-inflammatory markers. The switch from the high-fat to the standard diet resulted in the comparatively fast restoration of the baseline levels of the studied molecules. Although, the prolonged consumption of an HFD causes adipocyte hypertrophy in healthy male animals, the inflammatory process in VAT is well-coordinated, time-dependent, and reversible.

## 1. Introduction

White adipose tissue (WAT) consists mostly of adipocytes, surrounded by loose connective tissue. It also contains preadipocytes, macrophages, fibroblasts, and other cell types [1]. The major role of WAT is to store excess energy, but it also plays the role of an endocrine organ, synthesizing and secreting signaling molecules, specific for the adipocytes and named adipokines [1,2]. Some of the adipokines can trigger the synthesis and secretion of molecules like C-reactive protein (CRP), thus contributing to the development of inflammation [3,4].

The inflammatory effects of food have been well depicted by Nappo et al., who reported about transiently elevated levels of triglycerides, tumor-necrosis factor-α (TNF-α), interleukin-6 (IL-6), and soluble adhesion molecules, as a result of a single high-lipid ingestion [5]. The prolonged consumption of a high-fat diet (HFD) on the other hand, leads to the abnormal growth of the adipose tissue, the development of obesity, and an increased macrophage infiltration. This affects both the number of macrophages in the tissue and their phenotype. The activated macrophages secrete pro-inflammatory molecules such as TNF-α, IL-6, and monocyte chemoattractant protein-1, changing the overall secretion of the adipose tissue and leading to the development of inflammation [6,7]. Diet-induced inflammation is more pronounced in visceral adipose tissue (VAT) and is characterized by slightly elevated levels of the pro-inflammatory marker molecules, hence it is low-grade and chronic [8,9,10].

Although many studies have reported the development of low-grade, chronic inflammation among obese people and its connection with the development of many obesity-related diseases [6,8,11], there are insignificant data about the dynamics of the process, in its beginning. Our experiment attempted to evaluate a diet-induced inflammatory process in VAT [11] during its earliest stages, studying quantitative changes in pro- and anti-inflammatory marker molecules, CRP [12,13], serum amyloid-A (SAA) [14], and interleukin 4 (IL-4) [15].

## 2. Materials and Methods

### 2.1. Animal Care

The experimental design and all procedures were conducted in complete accordance with the requirements for the protection and humane treatment of laboratory animals, published under Directive 20/01.11.2013 by the Bulgarian Food Safety Agency (BAFS). This study had an approval for the usage of laboratory animals in this experiment (BAFS—resolution No. 55/23.06.2016) and it is in accordance with the ethical standards of the Medical University of Plovdiv (resolution of the University Ethics Committee—No. 1041/25.04.2017). All animals involved in the experiment were obtained from the vivarium of the Medical University of Plovdiv. All animals were kept under standard housing conditions in the vivarium, as follows: living space—350 cm^2^, temperature—22 ± 2 °C, humidity—55 ± 10%, and 12/12 h light/dark cycles. All animals had free access to food and water and were under the care of qualified personnel, including veterinarians. Our experiment did not require the usage of any aggressive reagents or substances that could increase suffering, death rate, or disturbance of the animals.

### 2.2. Study Design and Sample Collection

**Figure 1:**
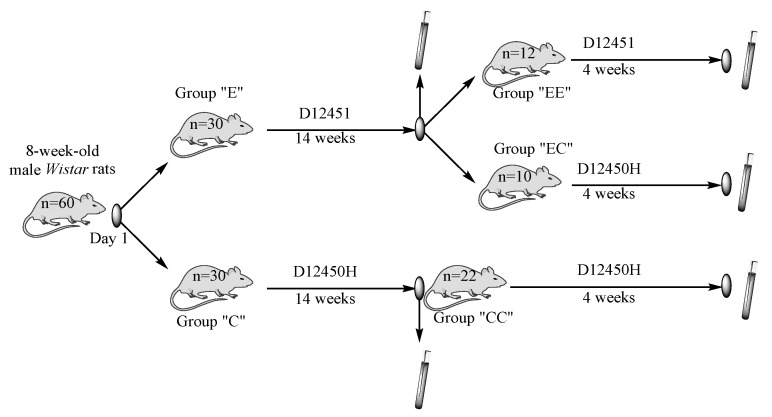
Study design and timeline of sample collection.

The criteria for animal selection included the following: 8-week-old male Wistar rats with a maximum birth interval of 2 days and a body weight of 135–165 g. We used two different types of rodent food in our experiment, standard “D12450H” and high-fat diet “D12451” (Research Diets, Inc., New Brunswick, NJ, USA). They were given to two experimental groups, with 30 experimental male Wistar rats in each; control group “C” were fed with D12450H and group “E” were fed with D12451 for a 14 week period. Eight animals were taken from every group, at random, on the 14th week and blood and tissue samples from VAT were collected. On the same week, the initial two groups were transformed into three new groups, as follows: The “C” group (*n* = 22) was our control group for the next 4 weeks and its label was changed to “CC”. The experimental group “E” was split into groups “EE” (*n* = 12), fed with the HFD and “EC” (*n* = 10), where the diet was replaced with the standard one for the next 4 weeks. The same sample collection procedure was performed on week 18. The study design and timeline of sample collection are illustrated in Figure 1. The sample size was calculated using a “minimal sample size for statistical significance” calculator. All animals who were not used and remained alive at the end of the experiment were returned back to the vivarium.

The details regarding the tissue sample collection have previously been described and published [16,17]. The tissue levels of CRP, SAA, and IL-4 were determined using the ELISA method and were visualized using immunohistochemical staining. The intensity of the staining was manually evaluated by two independent examiners. The expression of the markers was presented using a semiquantitative scale, where the staining intensity was categorized as follows: negative (−), weak intensity that corresponded to 25% stained cells (+), moderate—50% stained cells (++), and strong—75% stained cells (+++).

The body length and abdominal circumference were measured as follows: an animal was placed in a ventral position onto a plastic, non-extensible measuring tape and the measured naso–anal length was considered as body length. The abdominal circumference was assessed on the largest area of the rat abdomen.

### 2.3. Statistical Analysis

The collected data were statistically processed using SPSS v.19.0 (SPSS Inc., Chicago, IL, USA). The parametric data (weight, body parameters, and mass) were compared using a one-way ANOVA and Tukey’s test. The data are presented as mean ± SD. The non-parametric data (the levels of CRP, SAA, and IL-4) were compared using the Kruskal–Wallis test and, when a statistical difference was observed, the post hoc Dunn’s test for multiple comparison was applied. The data are presented as median (25th–75th percentile). Differences with *p*-values less than 0.05 were considered statistically significant.

## 3. Results

### 3.1. Body Weight, Body Mass Index (BMI), Relative Fat Mass (RFM), Conicity Index (CI), and Lee Index

The initial weight of the animals was 145 ± 15.26 g. Since we had only a few animals whose weight was between 125 and 135 g and only a few above 165 g, we compared the “C” and “E” groups, but no statistically significant difference was found. In addition, the comparison between the initial “C” and “E” groups in terms of BMI, CI, RFM, and Lee index did not show any statistically significant differences. Therefore, these data are not present in Table 1. The consumption of the HFD led to rapid growth in rats during the first 14 weeks of the experiment. The experimental group “E” showed a significantly higher weight, compared to the control group “C”, *p* = 0.004. The mean value of the body weight measured on week 18 was still the highest in the group that continued to ingest the HFD (group “EE”). However, this value was statistically insignificantly higher than the values for the body weight of the other two groups, “CC” and “EC”, *p* > 0.05. At the same time, the BMI for the “CC” group was higher compared to that of the “C” group, *p* = 0.003, showing that the control diet also contributed to the overall weight gain. We assume that the initial HFD intake predisposes the rats to additional weight gain, since the animals of both groups, “EE” and “EC”, had values much higher than those for group “E”, *p* = 0.002 and *p* = 0.042.

The highest BMI was measured for the “E” group, the animals from which were fed with the HFD for 14 weeks. The difference between groups “C” and “E” was statistically significant, *p* = 0.01. Despite the fact that the BMI for the “EE” group was still higher than the BMI of the “CC” and “EC” groups, there were no statistical differences detected, *p* > 0.05. The same dependence was observed for the CI. The highest value was detected for the “E” group, and it is much higher than the values of both the “C” (*p* = 0.006) and “CC” (*p* = 0.016) groups. Neither the Lee index nor the RFM showed any statistically significant differences.

The results for weight, BMI, RFM, CI, and the Lee index are shown in Table 1.

### 3.2. Qualitative Changes in the Levels of CRP in Tissue Homogenates from VAT

**Figure 2:**
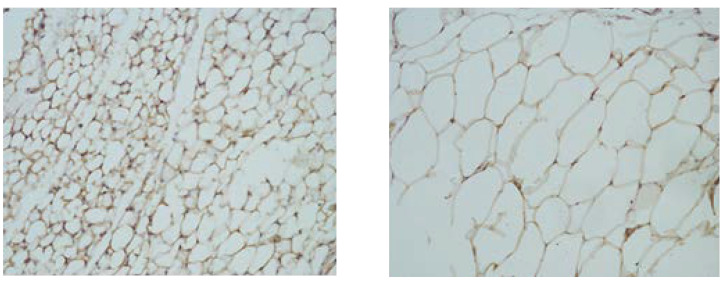
Microphotographs of CRP expression in visceral adipose tissue from rats fed with standard (**left**) and high-lipid diets (**right**). Magnification 40×.

The microphotographs of VAT samples (Figure 2) showed adipocyte hypertrophy in the groups subjected to the high-lipid diet. The intracellular distribution of CRP, detected using immunohistochemical analysis, was different in the experimental groups. It showed a clear tendency for an increase in the groups fed with the HFD, where the number of the positively stained cells was much higher (++), compared to the control groups (+).

**Figure 3:**
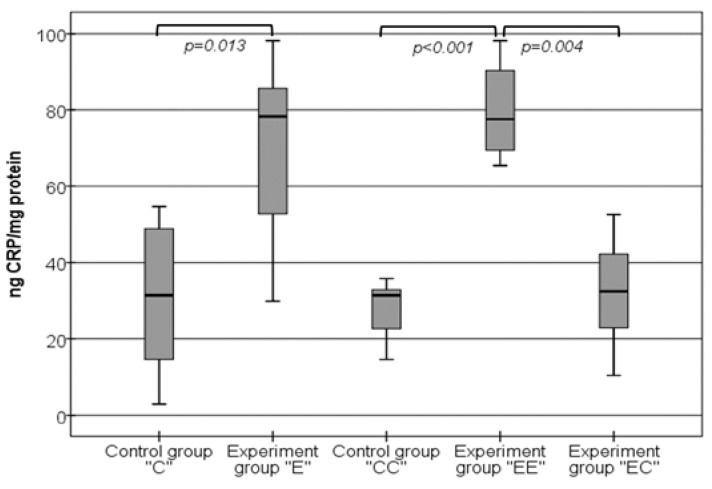
Changes in the levels of CRP in VAT homogenates.

These results were confirmed by the data obtained from the qualitative ELISA method, as shown in Figure 3. The levels of CRP in the experimental group “E” (78.31 ng/mg (52.03–86.81 ng/mg)) were significantly higher than the levels in the control group “C”, *p* = 0.013. The same dependence was observed when the experimental group “EE” (77.61 ng/mg (68.58–90.44 ng/mg)) was compared with the control group “CC” (31.44 ng/mg (20.72–33.61 ng/mg)), *p* < 0.001. The CRP levels in both control groups were almost the same, *p* > 0.05, which indicates that the additional period is not a reason for this pro-inflammatory factor to change. When both the “E” and “EE” groups were compared, the statistical analysis showed no further significant increase in the levels of CRP, *p* > 0.05. While the rate of the inflammatory response remained the same in the groups fed only with the HFD, the levels of CRP significantly decreased in the experimental group “EC”, where the diet was changed. We detected that the levels of CRP measured in this group, 32.44 ng/mg (22.9–44.05 ng/mg), were very similar to the values of the control groups (*p* > 0.05) and were significantly lower than the levels of the “E” and “EE” groups (*p* = 0.033 and *p* = 0.004, respectively).

### 3.3. Changes in the Levels of SAA in Visceral Adipose Tissue Samples

**Figure 4:**
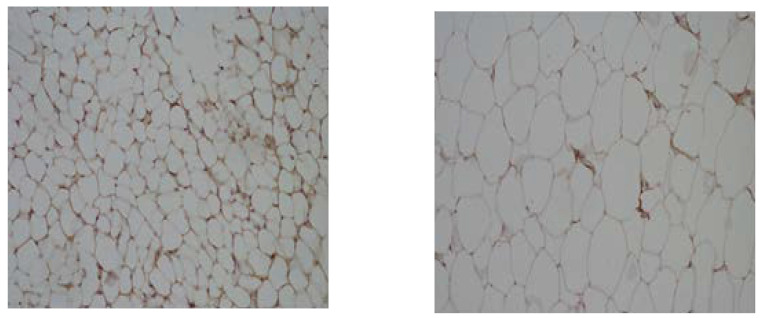
Microphotographs of SAA expression in visceral adipose tissue from rats fed with standard (**left**) and high-lipid diets (**right**). Magnification 40×.

The overall expression of the SAA protein showed almost the same pattern as the expression of CRP, as presented in Figure 4. It was lower in the groups fed with the standard diet (+) and higher in the groups that consumed the HFD (++).

**Figure 5:**
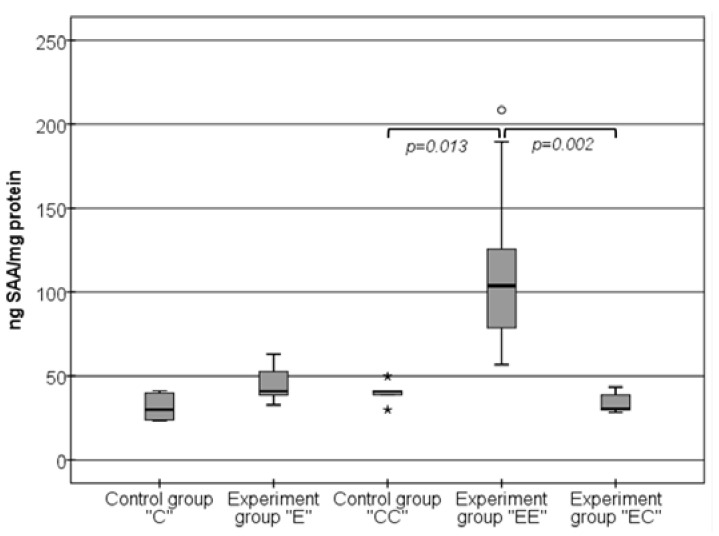
Changes in the levels of SAA in VAT homogenates. The symbols * and ^o^ state for outliers.

The ELISA method gave us additional and more detailed information about these changes, as presented in Figure 5. First, the levels of SAA, measured on week 14, were slightly higher in the experimental “E” group (40.93 ng/mg (37.62–54.41 ng/mg)) compared to the control “C” group (30.03 ng/mg (23.71–39.82 ng/mg)), but without a statistically significant difference, *p* > 0.05. However, this slight elevation of SAA in the “E” group was even more pronounced in the experimental group “EE”, which was fed with the high-lipid food for 18 weeks. The levels of SAA in this group, 103.76 ng/mg (78.13–141.61 ng/mg), were almost three times higher than the levels in both control groups “C” and “CC” (*p* < 0.001 and *p* = 0.013, respectively) and the “E” group (*p* = 0.002).

Our experiment established that the change in the food quality changed the levels of the pro-inflammatory SAA molecule. When the HFD was replaced by the standard one, the levels of SAA significantly decreased. The detected SAA levels in the “EC” group (30.47 ng/mg (29.84–39.21 ng/mg)) were almost the same as the levels of the “C”, “CC”, and “E” groups, *p* > 0.05, and much lower than the levels in the “EE” group, *p* < 0.001.

### 3.4. The Changes in the Levels of IL-4 in VAT Samples Follow the Same Pattern as the Changes in the Levels of SAA

**Figure 6:**
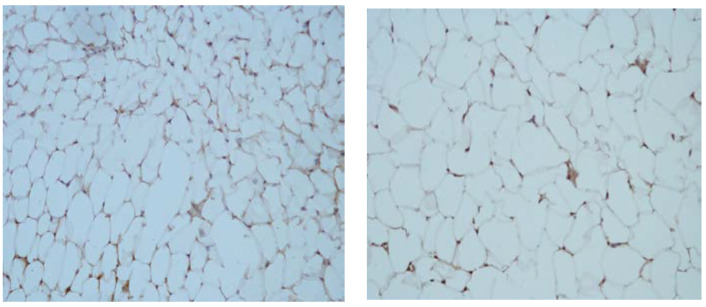
Microphotographs of expression of IL-4 in visceral adipose tissue from rats fed with standard (**left**) and high-lipid diets (**right**). Magnification 40×.

Figure 6 visualizes the immunohistochemical analysis, which showed no difference in the expression of IL-4 between the compared groups. The IL-4 expression was weak in the control groups, as well as in the group fed with the high-lipid diet.

**Figure 7:**
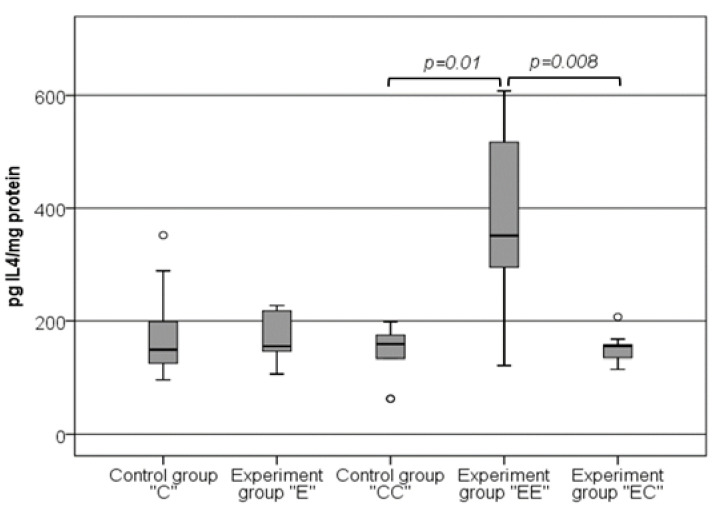
Changes in the levels of IL-4 in VAT homogenates. The symbol ^o^ states for outliers.

Figure 7 illustrates the changes in the levels of IL-4 in VAT homogenates. The detected levels of IL-4 were almost the same in both the control “C” (149.23 pg/mg (119.21–201.27 pg/mg)) and experimental “E” groups (155.44 pg/mg (137.92–220.22 pg/mg)), *p* > 0.05. In contrast to this result, we established more than a two-fold increase in the levels of IL-4 in the “EE” group, 351.68 pg/mg (283.57–519.09 pg/mg). The comparison of the data obtained in this group with groups “CC” (159.27 pg/mg (116.18–181.4 pg/mg)) and “EC” (155.69 pg/mg (133.85–160.77 pg/mg)) showed a clear statistical difference (*p* = 0.01 and *p* = 0.008, respectively). The results obtained in the control groups, as well as the experimental “E” and “EC” groups were similar and without any significant difference, *p* > 0.05.

## 4. Discussion

In this experiment, we studied body parameters and quantitative changes in inflammatory marker molecules to evaluate the effect of a high-fat diet on the development of obesity and low-grade, chronic inflammation in healthy organisms.

The prolonged intake of a HFD could lead to abnormal growth in VAT and provoke the development of low-grade, chronic inflammation [18]. This may result in the healthy tissue transforming into pathogenic fat depots [19]. The latter determines the necessity to make a design to estimate the adipose tissue abnormal growth, as well as to evaluate the risk for the development of inflammation and obesity-related disorders. One of the earliest markers for assessing general obesity that is applied in animal studies is the Lee index [20], although it has several limitations [21]. Another obesity marker is the conicity index. This assesses not only general obesity, but also tries to determine the body fat distribution [22]. Body mass index is the most widely used and promising marker to measure body fat and the risk for the development of obesity-related disorders, in many cases [23]. Nevertheless, CI appears to be the clearer marker in other studies [24,25]; thus, it can be used synergistically with the BMI. The relative fat mass index on third side provides a more indicative and precise assessment of the overall body fat distribution and the whole-body fat percentage. It also gives more accurate information about the connections between obesity and obesity-related disorders [26]. Therefore, each of the listed indices contributes to the overall evaluation of obesity and whole-body fat distribution and tries to predict the development of obesity-related disorders. Based on this information, we can state that the HFD used in our experiment causes a significant increase in the weight of the experimental animals. The same effect, but on mouse models, was also observed by Ji Y et al. [27]. Although we detected a significant difference in the BMI and CI at an earlier stage of the experiment, there is no other clear evidence for the development of obesity, abnormal adipose tissue growth, or for the atypical accumulation of body fats. Moreover, the initial fast weight gain and changes were followed by rapid adaptation to food and restoration of the body parameters.

One of the most specific visible markers of the inflammatory process in the adipose tissue is the presence of crown-like structures (CLSs). Their formation is due to the accumulation of macrophages, originating from newly recruited monocytes around dying adipocytes. Their number significantly increases in VAT in obesity [28]. The presence of CLSs in VAT is related to the activation of nuclear factor-κB (NF-κB), the increased expression of the inducible form of nitric oxide synthase, and the secretion of pro-inflammatory molecules such as IL-6 and TNF-α, thus promoting inflammation and obesity-related disorders [29,30]. Despite the well observed adipocyte hypertrophy, we did not detect any atypical or pathological accumulation of cells around them. We may conclude that, at this comparatively early stage of the HFD intake, the adipose tissue is still healthy and the inflammatory process is well-coordinated and organized.

The clinical laboratory analyses, however, showed that the inflammatory process is present in the tissue. In general, obesity is characterized by elevated serum CRP levels [12], as these levels show a positive correlation with adipocyte hypertrophy [13]. Under normal, physiological conditions, CRP is predominantly synthesized by the liver, although there is evidence for its synthesis and secretion by other cell types, like macrophages [31,32] and adipocytes [33]. This information was confirmed using the immunohistochemical results that show positively stained adipocytes. In the context of hypertrophic but still healthy adipose tissue, we assume that the main quantity of the marker is of adipocyte origin. This supports the hypothesis that macrophage infiltration is as a result of, rather than a consequence of, altered adipose tissue function [34]. However, our results contradict those of another study. Sjöholm et al. reported that the expression of CRP does not change in the adipose tissue of obese, but healthy, individuals [35], but can still be affected by pro-inflammatory cytokines [33]. This discrepancy may be due to the fact that we used animals in the current experiment. In addition, the detected changes in the CRP levels probably depend on intercellular, paracrine mechanisms between adipocytes and other cell types. However, such mechanisms were not the subject of our investigation and this could be considered as one of its limitations.

The levels of CRP are stable over a long period of time, show almost no circadian changes, and do not depend on the type of food ingested [36]. On the other hand, CRP accumulates at sites of inflammation or tissue damage [37]. Its elevated levels correspond either with the development of inflammatory process or they are assumed as a precondition for the development of disorders [36]. It is well established that CRP exhibits both pro- and anti-inflammatory effects, depending on its structure. The pentameric CRP has rather anti-inflammatory effects, whereas the monomeric has well-demonstrated pro-inflammatory features [38]. Another limitation of our experiment is that we did not determine the predominant form of CRP. Nevertheless, our data from the ELISA tests could be considered as further evidence for the development, or the result, of an inflammatory process in VAT.

The serum amyloid-A protein is involved in the removal of cholesterol from macrophages, thus accelerating the cholesterol efflux from damaged cells and tissues in areas with inflammation [39]. The levels of SAA are higher in obese individuals compared to in lean individuals, as well as to those who are in the process of losing weight [35]; hence, they depend on the nutritional status [40,41]. Another experiment demonstrated that a combined high lipid/high carbohydrate diet and an excess of cholesterol activate the expression of SAA in both the liver and adipose tissue [42]. Despite the lack of evidence about injured and inflamed sites within the adipose tissue, our experiment showed that a diet in which the caloric intake is determined by only an HFD also altered the levels of SAA. The effects can be depicted by a gradual increase in the tissue levels of SAA, dependent on diet duration. The short-term HFD consumption only tended to slightly elevate SAA levels, whereas the long-term HFD intake led to significantly higher SAA levels.

Another reason that causes the levels of SAA to increase is the rate of adipocyte hypertrophy, where metabolic disturbances also occur [14,40]. The increased secretion of SAA from hypertrophied adipocytes in obesity suggests that SAA is an active participant in the development of a local inflammatory process and probably links obesity with obesity-related disorders [43]. In our study, the prolonged consumption of an HFD led to visible adipocyte hypertrophy, even though there was no clear evidence for obesity. Therefore, we might speculate that, under these conditions, the only reason for the increased levels of SAA is an HFD and adipocyte hypertrophy. The increased quantity of SAA, along with the elevated CRP levels, could be interpreted as a developing inflammatory process in VAT, triggered by the diet [44].

Every inflammatory process, including low-grade and chronic inflammation, should be controlled by anti-inflammatory molecules. The origin and the levels of IL-4 in the adipose tissue mostly depend on the following two sources: natural killer T-cells type 1 (iNKT) and adipocytes. Normally, iNKTs are found in the adipose tissue of both humans and rodents. Their number is significantly decreased in obese patients, which affects the immune system, causing the development of inflammation [45]. The activated iNKTs shift the macrophage polarization towards the alternative (M2) phenotype, activating the IL-4/STAT6 signal cascade [27], thus reducing the inflammatory process. The same cascade exerts control over the abnormal development of fat depots, inhibiting adipogenesis and activating lipolysis [46]. Adipocytes, themselves, contribute to the local IL-4 levels, maintaining reciprocal crosstalk with the tissue macrophages and affecting the M1/M2 ratio [47]. The synthesis and secretion of IL-4 during our experiment was dynamic. The highest IL-4 levels were observed when the levels of both pro-inflammatory molecules were also significantly higher. Therefore, we might suggest that the anti-inflammatory IL-4 secretion was not yet impaired. Moreover, our results are in concordance with earlier experiments that have shown that the levels of IL-4 increase during dietary-induced obesity [48] and decrease with weight loss [49].

An interesting parallel could be drawn between our findings and the study conducted by Poret et al. They demonstrated that the obesity-prone rats (Osborne-Mendel, OP) preferred an HFD, consumed more fats, gained more weight, and developed higher levels of inflammation, whereas Wistar rats had no preferences to diet, but still developed inflammation when fed with an HFD. On the other hand, the anti-inflammatory phenotype examined by our experiment resembles the response of obesity-resistant rats (S5B/Pl, OR), when they are randomly assigned to either high- or low-fat diets [50]. Interestingly, in our experiment only the adipocyte size, rather than macrophage infiltration, followed the same pattern as in OR rats, even though we detected an inflammatory process. It is well known that the levels of both CRP and SAA strongly correlate with adipocyte size [13,40]. Hence, we might speculate that the observed adipocyte hypertrophy was the main reason for the CRP and SAA elevation. Although the interactions between adipocytes and macrophages are critical in determining the overall inflammatory process in the tissue, adipose tissue inflammation is far more complex than simply being a function of macrophage infiltration [51].

The time-dependent changes in the inflammatory response are of key importance for its progression to a chronic and pathological process. This could be partially explained by the epigenetic effects of food. The intake of an HFD, especially rich in saturated, long-chain fatty acids (SFAs), leads to elevated levels of inflammatory marker molecules, mostly as a result of the activation of signaling pathways, triggered by Toll-like receptors (TLR-4) [51,52]. TLR-4 may also play the role of a “sensor” for endogenous lipids, contributing to the development of obesity-related disorders [53]. The activation of the receptor by SFAs leads to inflammatory changes in both macrophages and adipocytes, activating NF-kB [54] and increasing the gene expression and secretion of adipokines [55]. SFAs from the diet also upregulate the gene expression of the pro-inflammatory TNF-α. TNF-α is one of the activators of the CRP [56] and SAA gene expression rates [14]. On the other hand, TNF-α increases the rate of lipolysis, contributing to the locally increased levels of the fatty acids that are secreted by adipocytes. Both the endogenous and dietary-delivered fatty acids additionally induce the secretion of TNF-α from the tissue macrophages, further increasing the rate of the inflammatory process [57]. TNF-α has also been associated with the development of adipose tissue fibrosis in obese mice [58].

While the levels of CRP appeared to be elevated at a much earlier stage of our experiment, the levels of SAA and IL-4 required more time to increase in VAT. However, macrophage polarization, the effects of TLR-4, and the levels of TNF-α were not studied in this experiment. Despite these additional limitations, the changes in the levels of CRP and SAA in VAT are indicative of a developing, dietary-induced inflammatory process. Since the levels of both pro- and anti-inflammatory molecules increased over time, we assume that an HFD provokes inflammation, but the normal homeostasis of the tissue is not disrupted yet, at this early stage.

## 5. Conclusions

Our results showed that the high-fat diet barely affected obesity indices, whereas it induced vascular adipocyte hypertrophy and triggered development of inflammation in healthy male rats. Despite the lack of clear evidence of obesity, the inflammatory process proceeded further with the increase in the levels of pro-inflammatory molecules, resembling the inflammatory process in obesity. However, the anti-inflammatory markers also increased gradually, possibly counteracting the progression of the inflammation. We might speculate that the high-fat diet may provoke its adverse effects on visceral adipose tissue, causing the development of obesity-associated diseases, but after its consumption for a much longer period. Moreover, we demonstrated that the switch from the high-fat to the standard diet resulted in a comparatively fast restoration of the baseline levels of the studied molecules. Therefore, within the limitations of this study, we can conclude that the diet-induced inflammatory process in visceral adipose tissue is well coordinated, time dependent, and reversible at an early stage in healthy organism.

## Figures and Tables

**Table 1:** Body weight, body mass index, Lee index, conicity index, and relative fat mass of male Wistar rats, divided in groups according to their diet.

	*n*	Weight (g): Mean ± SD	BMI (kg/m^2^) Mean ± SD	Lee Index (g/mm) Mean ± SD	CI (kg/m) Mean ± SD	RFM (kg/m) Mean ± SD
Initial	60	145.42 ± 15.26	–	–	–	–
Group “C”	30	295.88 ± 31.88	7.26 ± 0.74	329.80 ± 14	1.89 ± 0.15	40.3 ± 1.06
Group “E”	30	321.50 ± 24.02 **	7.85 ± 0.87 *	332.05 ± 20.62	2.18 ± 0.15 **^#^	40.52 ± 1.31
Group “CC”	8	331.38 ± 27.51	6.45 ± 0.32	306.92 ± 5.77	1.92 ± 0.13	38.37 ± 1.1
Group “EE”	8	360.13 ± 23.30	7.03 ± 0.65	317.16 ± 9.42	2.05 ± 0.07	39.41 ± 1.11
Group “EC”	8	341.63 ± 51.60	6.80 ± 0.28	313.59 ± 6.18	1.98 ± 0.15	38.95 ± 0.64

Significance levels are marked as follows: 1. * *p* < 0.05 and ** *p* < 0.01—“E” group versus “C” group; 2. ^#^ *p* < 0.05—“E” group versus “CC” group.

## Data Availability

All data are available on demand.
